# Are food and beverage purchases reflective of dietary intake? Validity of supermarket purchases as indicator of diet quality in the Supreme Nudge Trial

**DOI:** 10.1017/S0007114524002630

**Published:** 2024-11-28

**Authors:** Chiara Colizzi, Josine M. Stuber, Yvonne T. van der Schouw, Joline W. J. Beulens

**Affiliations:** 1 Department of Global Public Health and Bioethics, Julius Center for Health Sciences and Primary Care, University Medical Center Utrecht, Utrecht University, Heidelberglaan 100, 3584 CX, Utrecht, the Netherlands; 2 Amsterdam UMC location Vrije Universiteit Amsterdam, Epidemiology and Data Science, De Boelelaan 1117, Amsterdam, the Netherlands; 3 Amsterdam Public Health, Amsterdam, the Netherlands; 4 Upstream Team, Amsterdam UMC, De Boelelaan 1117, Amsterdam, the Netherlands

**Keywords:** Diet quality, Purchase data, Dietary assessment, Supreme Nudge Trial, Nutritional epidemiology

## Abstract

Dietary intake assessment is often complicated by intrinsic bias. This study investigated whether food purchase data could constitute a valid indication of dietary intake, by evaluating the extent to which diet quality as measured by supermarket food purchases is correlated with diet quality as measured by reported dietary intake. We used data from the Supreme Nudge cluster-randomised controlled supermarket trial (*n* 227). Data were collected at baseline from supermarket purchases (loyalty cards) and a dietary questionnaire (short forty-item FFQ) to compute two scores reflecting diet quality from purchasing data (purchased diet quality) and FFQ (consumed diet quality). Both scores constituted thirteen food groups and could theoretically range from 0 (low diet quality) to 130 (high diet quality). The relationship between purchased diet quality and consumed diet quality was assessed using correlation coefficients and the Bland–Altman limits-of-agreement method. Multiple linear regression was fitted between purchased diet quality and consumed diet quality, adjusted for age, sex, waist circumference, educational level and household size. Consumed and purchased diet qualities were modestly positively correlated (Pearson’s ρ = 0·31, 95 % CI 0·18, 0·42). A positive association from linear regression was found after confounding adjustments (β_baseline_ = 0·22, 95 % CI 0·10, 0·34). The purchased diet quality was systematically lower than the consumed diet quality. This study found that diet quality as measured by supermarket purchases provided a reasonable indication of diet quality as reported by short-FFQ, albeit modest.

Diet quality is an important modifiable risk factor for chronic diseases, including CVD and type 2 diabetes^(1,2)^. Dietary choices are often guided by automatic cognitive processes, rather than by conscious decision-making^(3)^. There is, therefore, a growing interest in interventions that could help individuals improve their diets by targeting their food purchasing behaviour using subconscious strategies, such as nudging (e.g. placing fruits at the check-out counters instead of unhealthy snacks) and pricing strategies (e.g. lowering costs of fruits and vegetables by using subsidies or increasing prices of unhealthy snacks or sugar-sweetened beverages via sugar taxes)^(4–7)^.

Interventional studies in point-of-choice settings, such as supermarkets and restaurants, often rely on purchasing data to measure intervention effects^(6,8)^. These data are cost-effective and faster to collect than traditional dietary assessment methods, especially in large-scale intervention studies or in settings where it might not be feasible to collect reported intakes^(5)^. However, purchase data also present some challenges; for instance, household food shopping is often done by one family member and might therefore not reflect individual diet. Additionally, relying solely on one supermarket chain’s loyalty card to collect food purchasing data does not capture purchases at other food retailers outside the study supermarkets. Also, purchase data do not account for food waste at home^(9,10)^. Although purchasing data do not directly translate to individual dietary intakes, they have the potential to be an adequate proxy for diet quality^(11)^.

Only a few studies have evaluated the correlation between food and beverage purchases and diet quality, which are difficult to compare due to different assessment methods of diet quality and purchasing data^(9,10,12,13)^. For instance, two studies found a moderate correlation between purchases and diet quality expressed with Healthy Eating Index-2010 scores^(9,13)^. One study used a revised Healthy Purchase Index to assess diet quality of food purchases and found it was strongly correlated with several indicators of nutritional quality, but not with total energy content^(12)^. To our knowledge, only the study by Appelhans *et al.* (2017) used the same indicator to measure diet quality from both purchases and food consumption (the Healthy Eating Index-2010)^(9)^, and another study, by Parker *et al.* (2021), measured the correlation between specific food groups purchased and consumed^(13)^.

Overall, the evidence on the use of food purchases as indicators of diet quality is still quite limited and underscores the need for more validation studies. Knowing the validity of purchasing data could have important implications for epidemiological studies. Not only because purchasing data is more cost-efficient to collect than traditional dietary measurements, but it could also have the potential to be used to evaluate large-scale population-level interventions, such as sugar taxes or food assistance programmes^(14)^. For these reasons, it is relevant to study the extent to which food purchases are a reflection of dietary quality, including differences across food groups and the demographic characteristics that may affect this relationship.

## Methodology

### Study design

This study is a secondary analysis of a 12-month cluster-randomised controlled supermarket trial – the Supreme Nudge trial – aimed at improving lifestyle behaviours and lower cardiometabolic disease risk in adults living in lower socio-economic position neighbourhoods in the Netherlands. The study was conducted in twelve supermarkets located in a lower socio-economic position neighbourhood, and it evaluated the effects of supermarket nudging and pricing strategies promoting healthy products and of a mobile physical activity app promoting walking behaviours^(15,16)^. Participants were followed for 6 (*n* 4 supermarkets) to 12 months (*n* 8 supermarkets), ensuring enough time to capture purchasing habits. Data were collected at baseline and after 3, 6 and 12 months of follow-up. The present study uses the baseline data only, to avoid any potential influence from the nudging and pricing interventions on the participants’ shopping behaviour.

### Study population

**Figure 1.**
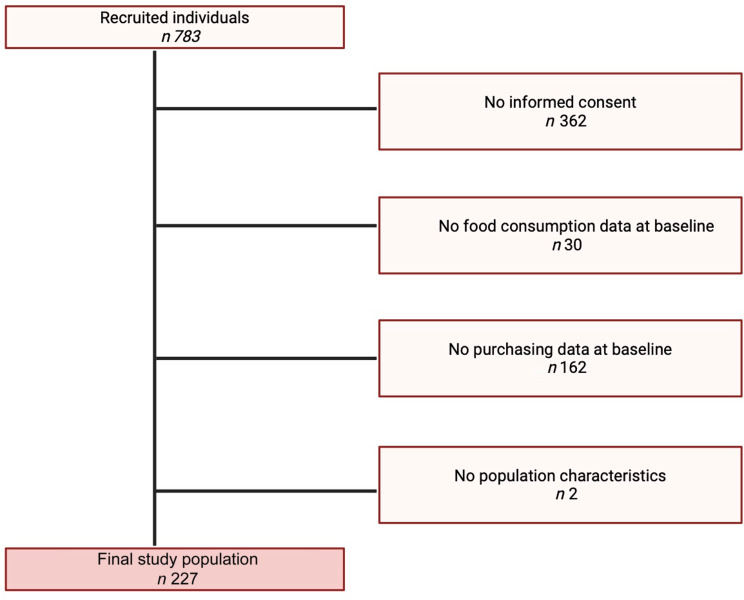
Study population flow chart.

The study population is constituted by the participants of the Supreme Nudge Trial^(15,16)^. The trial was conducted among regular supermarket customers who bought more than half of their weekly groceries at the participating supermarkets, to ensure that the participants’ purchases reflected most of their overall food purchases^(15)^. Figure 1 shows the flow chart of the study population. The final study population included 227 individuals (Fig. 1).

Household weights were applied to the study population to calibrate the study population to the general Dutch population. The weights for one-person household were calculated by dividing the percentage of one-person household in the Netherlands by the percentage of one-person household in the study population. The same was done to calculate the weights for multi-adults households. Information on household size in the Netherlands is openly available on Statistics Netherlands^(17)^ and reflects whether a participant belonged to a one-person household or a multi-adults household.

### Ethical approval

The original study protocol complies with the principles of the Declaration of Helsinki and was approved by the Medical Ethics Review Committee of VU University Medical Center in Amsterdam, the Netherlands (reference no. 2019.334). All participants provided written informed consent prior to study enrolment.

### Data collection

Information on diet quality was collected via the Dutch Healthy Diet 2015 FFQ, which is a short-validated forty-item FFQ^(18)^. The questionnaire asks participants about their dietary intake during the previous month and reflects adherence to the Dutch dietary guidelines of 2015^(19)^. Adherence to the guidelines is measured with a score from 0 to 150 for fifteen food groups, with 0 indicating no adherence and 150 reflecting the highest adherence (Dutch Healthy Diet 2015-index (DHD15-index) scores)^(18)^.

Purchase data were collected using the supermarket’s customer loyalty card, and participants were instructed to use the loyalty card at each supermarket visit. Each purchased product was classified into overarching product groups (e.g. ‘dairy products’) and further categorised into healthy (recommended in the Dutch dietary guidelines) and unhealthy (not recommended) products within product groups^(19)^. The average number of grams purchased was calculated per block of 4 weeks for each product group. In this study, all the grams of food purchased were divided by 28, to reflect food purchases in grams per d.

### Assessment of diet quality

Diet quality from the FFQ and from purchases was classified using the DHD15-index. In this study, we referred to consumed diet quality when the DHD15-index reflected diet quality based on reported intakes from the FFQ and purchased diet quality when the DHD15-index reflected diet quality derived from purchasing data.

Both scores followed the DHD15-index’s guidelines and cut-off points described by Looman *et al.* (2015)^(18)^ but with some alterations: red and processed meats were combined into one group, as well as tea and coffee. For these food groups, the food categories used to classify purchased food items did not match the food group components used by Looman *et al.* ^(18)^, as these food items had already been combined into the same food groups in the purchasing data. Salt was excluded from the original scoring as it could not be accounted for separately from the purchasing and consumption data. Sweet and salty snacks were added as an additional scoring component.

Each component could have a minimum score of 0 points and a maximum score of 10 points (online Supplementary Table 1). Consumed diet quality and purchased diet quality each consisted of thirteen food groups, and the final score could range from 0 (low diet quality) to 130 (high diet quality). Both indices included the following food groups: fruit, vegetables, whole grains, legumes, nuts, fish, dairy products, fats, red and processed meats, sugar-sweetened beverages, alcohol, coffee and tea and snacks. Based on the guidelines from Looman *et al.*, consumed and purchased diet qualities were operationalised using adequacy components for fruit, vegetables, whole grains, legumes and nuts. For these food groups, the adequacy components ensured that higher intakes resulted in higher scores. Moderation components scored individuals with a high score for low intakes and a low score for high intakes. Moderation components were used for red and processed meats, snacks, sugar-sweetened beverages and alcohol intake. The fat group was calculated using a ratio between unsaturated and saturated fats, and dairy products were calculated using an optimum component. Tea and coffee were a quality component, based on the type of coffee (filtered or unfiltered), according to studies showing that unfiltered coffee may be linked to increased levels of cholesterol^(20)^. For this component, no consumption of unfiltered coffee and drinking more than three cups of tea a day was awarded 10 points, whereas any consumption of unfiltered coffee was assigned 0 points^(18)^.

Purchased diet quality was further adjusted for participants’ shopping patterns, to partially account for the fact that purchases made at a participating study supermarket might have not reflected a person’s entire food shopping pattern. Participants were asked about the frequency of shopping at stores other than the study supermarket, which included bakeries, fish shops, greengrocers, butchers, other supermarkets or online supermarkets. For each of these other food retailers, data were dichotomised into a binary variable: 0 = those who never shopped at any other store, and 1 = those who shopped at another store at least once in the past 2 weeks. This binary variable was used to calculate the percentage multipliers. Online Supplementary Table 2 shows the percentages of individuals shopping at least once at another food retailer per time point. Percentage multipliers were applied to those who shopped at least once in the previous 2 weeks at another retailer. Different percentage multipliers were applied to different food groups: the percentage multiplier for purchases at a bakery was applied to whole-grain products and sweets; shopping at a butcher to red and processed meat; shopping at a greengrocer to fruit and vegetables; shopping at a fish shop to fish; and finally all other food groups were adjusted for the percentage of shopping done at any other food retailer (e.g. farmers market, local market, online supermarket and other supermarkets), as an average. For example, for the whole-grain component, the adjustment for this score component was done based on the following formula:

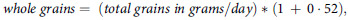




where 0.52 represents the 52 % of the sample that shopped at least once in the past 2 weeks at a bakery.

### Covariates

A baseline questionnaire collected information on several shoppers’ characteristics, including age (in years), sex (males, females), waist circumference (in cm), educational attainment and household size^(15)^. Educational attainment was categorised into low (no education or primary education), medium (secondary education) and high (tertiary education). Waist circumference was measured by the participant at home, via a measurement tape with an instructional video. Lastly, household size was reported in number of adults and number of children per household. In this study, number of adults per household was used to determine whether the participant belonged to a single or multi-adult household. More information on the baseline characteristics of the study participants can be found elsewhere^(15)^.

### Statistical analysis

Statistical analyses were conducted in SPSS 28.0.0.0 (190), and the α threshold for significance was set at *P*< 0·05. Descriptive statistics were calculated to characterise the study sample and food consumption variables. Variable distribution was examined using histograms and normal quantile plots. Population characteristics were described at baseline, using mean and sd for normally distributed continuous variables, median and interquartile range for skewed continuous variables and count and percentage for categorical variables.

Depending on the distribution of the variables, Pearson’s or Spearman’s rank correlation coefficients (ρ) were used to assess the correlation between consumed diet quality and purchased diet quality. The correlation was also tested in the individual food groups of both diet quality scores. A multiple linear regression was also fitted between purchased diet quality and consumed diet quality, with adjustments for age, sex, waist circumference, educational level and household size. The interaction was tested between purchased diet quality and age, sex and educational level. When the interaction was statistically significant (*P* < 0·05), stratified analysis by subgroups was conducted.

The Bland–Altman limits-of-agreement method was used to examine the agreement between consumed diet quality and purchased diet quality, to determine the extent to which purchased diet quality provides an unbiased estimate of consumed diet quality across the range of observed scores^(21,22)^. The Bland–Altman method quantifies the mean differences in the estimates as bias and provides 95 % confidence intervals for the difference^(21)^. Additional analyses were conducted to examine moderators of agreement between consumed diet quality and purchased diet quality. For this analysis, the difference between consumed diet quality and purchased diet quality was calculated, and then correlation was used to investigate the relationship between the difference in diet quality scores and the following potential moderating factors: age, sex, educational level, household size and waist circumference. For continuous variables, Pearson’s correlation was used, for dichotomous variables (e.g. sex), the point-biserial correlation coefficient, while for categorical variables (e.g. educational level), Spearman’s correlation was used. As a sensitivity analysis, the correlation analyses were repeated without adjusting for shopping at other stores.

## Results

The study population of 227 individuals at baseline were on average 58 (sd 10) years of age, were mostly female (75 %) and were either living alone (40 %) or with a partner (50 %) and largely without any children (74 %) (Table 1). The mean consumed diet quality was 87 (sd 15), while the mean purchased diet quality was 53 (sd 16) (Table 2). While only 24 and 26 % of participants visited a greengrocer or a butcher at least once, respectively, 93 % of the participants visited another food retailer at least once in the last 2 weeks (Table 1).

**Table 1.** Population characteristics at baseline (*n* 227) (Numbers and percentages; mean values and standard deviations)

Population Characteristics*	Mean	SD
Age	58	10
Sex (*n*, %)		
Males	57	25
Females	170	75
Waist circumference, in cm		
Males	102	10
Females	94	13
Educational level (*n*, %)		
Low	56	25
Medium	87	38
High	85	37
*n* of adults per household (*n*, %)		
1	91	40
2	113	50
3	18	8
4	5	2
*n* of children per household (*n*, %)		
0	168	74
1	21	9
2	28	12
3	9	4
4	1	0·4
Visited a fish store at least once in the past 2 weeks (*n*, %)		
No	135	60
Yes	92	41
Visited a butcher at least once in the past 2 weeks (*n*, %)		
No	169	74
Yes	58	26
Visited a greengrocer at least once in the past 2 weeks (*n*, %)		
No	172	76
Yes	55	24
Visited a bakery at least once in the past 2 weeks (*n*, %)		
No	111	49
Yes	116	51
Visited any other food retailer (online, local market, farmers market, other supermarkets) at least once in the past 2 weeks (*n*, %)		
No	16	7
Yes	211	93

* Expressed as mean and sd, unless stated otherwise.

**Table 2.** Diet quality in the population at baseline (*n* 227) (Mean values and standard deviations; median values and interquartile ranges)

Food consumption^*^	Mean	sd
Consumed diet quality, total, scored 0 (low adherence) to 10 (high adherence)	87	15
Consumed diet quality, by sub-components, scored 0 (low adherence) to 10 (high adherence)		
Vegetables	6	3
Fruit	7	3
Whole grains	8	2
Legumes	9	2
Nuts, median, IQR	7	3–10
Dairy products	6	3
Fish	7	3
Added fats, median, IQR	10	2–10
Tea and coffee	7	2
Red and processed meat	8	2
SSB	9	2
Alcohol	9	2
Snacks	7	3
Purchased diet quality, total, scored 0 (low adherence) to 10 (high adherence)	53	16
Purchased diet quality, by sub-components, scored 0 (low adherence) to 10 (high adherence)		
Vegetables, median, IQR	4	1–9
Fruit, median, IQR	3	1–9
Whole grains, median, IQR	2	0–6
Legumes, median, IQR	0	0–10
Nuts, median, IQR	0	0–0
Dairy products, median, IQR	0	0–6
Fish, median, IQR	0	0–8
Added fats, median, IQR	0	0–1
Tea and coffee, median, IQR	0	0–10
Red and processed meat, median, IQR	4	0–8
SSB, median, IQR	7	2–10
Alcohol, median, IQR	10	0–10
Snacks, median, IQR	8	5–10

SSB, sugar-sweetened beverages.

* Expressed as mean and sd, unless stated otherwise.

Purchased and consumed diet qualities were modestly positively correlated (Pearson’s ρ = 0·31, 95 % CI 0·18, 0·42). With regard to the correlation by score component, several food groups showed very low correlations between purchases and intakes such as vegetables (ρ_baseline_ = 0·06, −0·07, 0·20), legumes (ρ_baseline_ = 0·03, −0·13, 0·18), dairy products (ρ_baseline_ = −0·06, −0·19, 0·08) and tea and coffee (ρ_baseline_ = −0·04, −0·17, 0·10). The strongest and statistically significant correlations were found for fruit (ρ_baseline_ = 0·16, 0·02, 0·29), nuts (ρ_baseline_ = 0·20, 0·06, 0·33), fish (ρ_baseline_ = 0·18, 0·04, 0·31), red and processed meat (ρ_baseline_ = 0·18, 0·05, 0·31) and alcoholic beverages (ρ_baseline_ = 0·38, 0·26, 0·50) (Table 3). Moreover, the correlations were lower per single score component than for the total diet quality indices, with the exception of alcoholic beverages.

**Table 3.** Pearson’s and Spearman rank’s correlation coefficients between purchased and consumed diet qualities at baseline, overall and by score component (Correlation coefficient and 95 % confidence intervals)

	Correlation coefficient ρ	95 % CI
Overall^†^	0·31	0·18, 0·42^**^
Score components^‡^		
Vegetables	0·06	–0·07, 0·20
Fruit	0·16	0·02, 0·29^*^
Whole grains	0·13	–0·01, 0·26
Nuts	0·20	0·06, 0·33^**^
Legumes	0·03	–0·13, 0·18
Dairy products	–0·06	–0·19, 0·08
Fish	0·18	0·04, 0·31^*^
Red and processed meat	0·18	0·05, 0·31^**^
Fats	–0·04	–0·19, 0·10
Tea and coffee	–0·04	–0·17, 0·10
SSB	0·21	0·07, 0·34^**^
Snacks	0·01	–0·16, 0·19
Alcohol	0·38	0·26, 0·50^**^

SSB, sugar-sweetened beverages.

* Significant at 0·05.

** Significant at 0·01.

† Calculated using Pearson’s correlation.

‡ Calculated using Spearman’s rank correlation.

**Figure 2.**
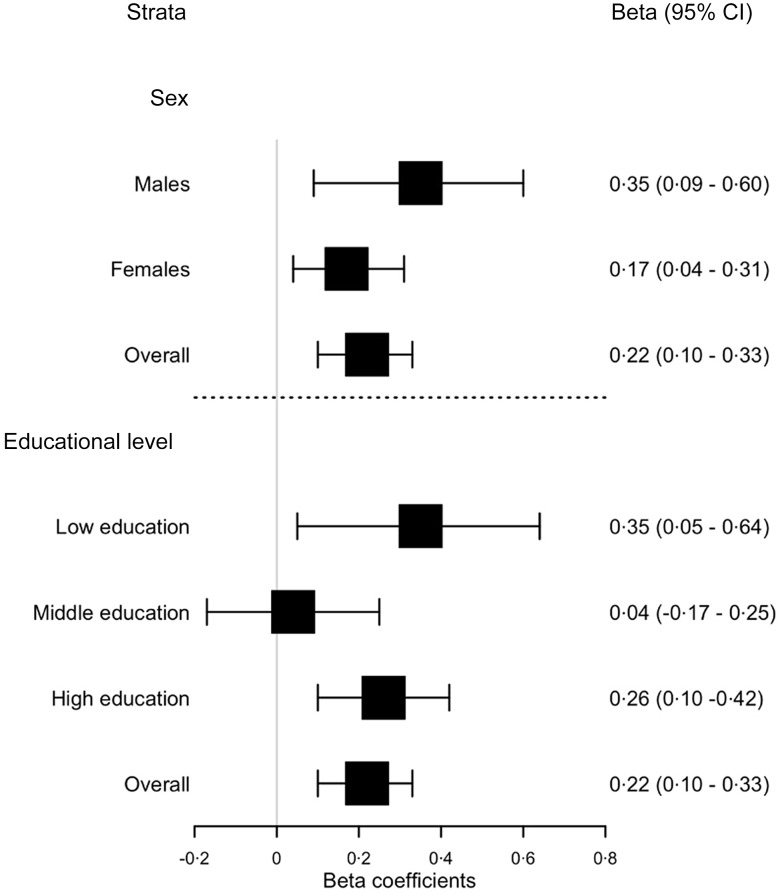
Association between purchased diet quality and consumed diet quality, stratified by sex and educational level †. † Adjusted by age, sex, waist circumference, educational level and household size.

Multiple linear regression showed that the purchased diet quality was statistically significantly associated with consumed diet quality, also after adjusting for demographics, albeit slightly attenuated compared with the crude model (β_baseline_ = 0·22, 95 % CI 0·10, 0·34) (Table 4). Stratified analyses were conducted for males and females and across levels of educational attainment (Fig. 2). The forest plot shows that the association between purchased diet quality and consumed diet quality was the strongest among males (β = 0·35, *P* = 0·01) and among those with the lowest (β = 0·35, *P* = 0·02) and highest educational level (β = 0·26, *P* < 0·001).

**Table 4.** Multiple linear regression for the association between purchased diet quality and consumed diet quality (*n* 227) (Beta coefficient and 95 % confidence intervals)

Outcome	Beta coefficient	95 % CI	*P*
Consumed diet quality			
Crude model	0·28	0·16, 0·39	< 0·001
Model 1^*^	0·22	0·10, 0·34	< 0·001

* Adjusted by age, sex, waist circumference, educational level and household size.

**Figure 3.**
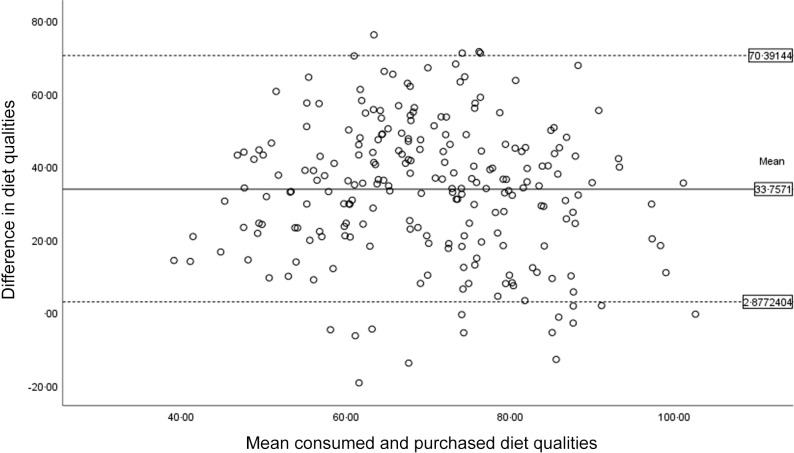
Bland–Altman’s plot of limits of agreement between consumed diet quality and purchased diet quality. Difference in diet quality refers to consumed diet quality – purchased diet quality.

Purchased diet quality was systematically lower than consumed diet quality. The Bland–Altman plot showed a mean difference between purchased and consumed diet qualities of 33·76 (Fig. 3). The mean difference did not appear to be linked to the level of intake but was equal across the indices. Discrepancies between purchased and consumed diet qualities were unrelated to age, sex, educational level, household size and waist circumference (Table 5).

**Table 5.** Correlation between the difference in purchased and consumed diet qualities and potential moderators (*n* 227) (Correlation coefficient and 95 % confidence intervals)

	Correlation coefficient, ρ	95 % CI
Difference in diet quality scores		
Age^*^	–0·06	–0·19, 0·07
Sex^†^	–0·09	–0·22, 0·04
Waist circumference^*^	–0·06	–0·19, 0·07
Educational level^‡^	0·08	–0·06, 0·21
Household size^‡^	–0·13	–0·26, 0·004

* Calculated using Pearson’s correlation.

† Calculated using point-biserial correlation.

‡ Calculated using Spearman’s rank correlation.

Sensitivity analyses did not alter the main findings. Baseline purchased diet quality and correlation analysis without adjustment for other stores showed similar results, except for the correlation for the dairy product component (ρ_baseline_ = 0·21, 0·07, 0·34) (online Supplementary Tables 3–5).

## Discussion

This study found that diet quality as derived from loyalty-card supermarkets’ purchases had a moderate agreement with diet quality as derived by reported dietary intake via a short forty-item FFQ. In stratified analyses, a stronger association was found for males compared with females and in those with the lowest and highest level of education, compared with those with a medium level of educational attainment. Additionally, purchased diet quality of fruits, nuts, fish and alcohol had the highest and most significant correlation with their corresponding consumed food group. Purchased diet quality was systematically lower than the consumed diet quality, although differences were unrelated to age, sex, educational level, waist circumference or household size.

This study has several limitations that need to be addressed. First, the sample size was relatively small, and study samples at baseline and follow-up comprised different numbers of people. Moreover, the study assessed diet quality using a short forty-item FFQ, which is a relatively imprecise measurement of diet quality on an individual level, compared with the assessment of diet quality through 24-h recalls or a complete FFQ. Additionally, agreement between food purchases and dietary intake is also affected by the bias intrinsic to FFQ. Indeed, although widely accepted as a valid measurement of dietary intake, they are not without limitations, such as recall bias and social desirability bias^(23)^. Moreover, because of a lack of available data on salt intake, type of meat and tea and coffee as separate food items, the diet quality scores had to be slightly modified by excluding the salt group and grouping together the red and processed meat components and the tea and coffee components, which were separated and scored differently in the original DHD15-index^(18)^. Lastly, no data were available on other demographic and lifestyle factors, such as household income, BMI and energy intake, which could have been of interest in the relationship between diet quality derived by reported food intake and diet quality derived by supermarket purchases^(8,9)^. Thus, possible residual confounding cannot be excluded.

The main strength of this study relates to the original study design. The participants had been recruited using a wide range of recruitment strategies from socially disadvantaged neighbourhoods, with the aim of including a relatively large number of individuals with a low educational level^(15,16)^. This likely increased the diversity in the study population and may have partially accounted for the lack of information on other determinants of social inequality. Also, all participants indicated to do at least 50 % of their shopping at the participating supermarket, reducing to some extent the influence of shopping at other stores on the overall food purchases^(15,16)^. Nonetheless, it was not possible to quantify the amount of foods purchased at other food retailers and therefore to account for the effect that these purchases may have had on individual diet quality. Because the relationship between purchases and individual diet was limited by the choice of including only those who did more than 50 % of the shopping at the participating supermarkets, it cannot be generalised to other studies that might include all customers. Another strength is that diet quality was measured with the same indicator and using the same scale for both reported dietary intake and purchase data. Additionally, this research tried to account for the household composition and shopping behaviour of the participants, which allowed for more precise estimates of consumed and purchased diet quality at the individual level.

While no other studies have, to our knowledge, measured the association between food purchases and dietary intake using the DHD15-index as a measure of diet quality, these findings are in line with other studies that have assessed this relationship using different indicators^(9,11–13)^. Perignon *et al.* (2023) used a revised Healthy Purchase Index, and Parker *et al.* (2021) used the Grocery Purchase Quality Index to measure the nutritional quality of food purchases^(12,13)^, while Appelhans *et al.* (2017) measured diet quality using nutrient densities^(9)^. Compared with this study, Parker *et al.* (2021) also found moderate agreement between mean Grocery Purchase Quality Index and Healthy Eating Index-2010 scores derived from FFQ (baseline *r*= 0·41; 3 months *r*= 0·31, *P*< 0·001)^(13)^. Appelhans *et al.* (2017) used the Healthy Eating Index-2010 and found moderate concordance (ρc = 0·57, *P*< 0·0001) and minimal bias between food purchases derived from collected receipts and dietary intake derived by 24-h recalls^(9)^. The use of 24-h recalls, instead of FFQ, might explain higher agreement between food purchases and food consumption in the study by Appelhans *et al.* (2017) than in this study.

The strength of the correlation between diet quality scores varied across food groups, albeit correlations were weaker across all food groups (except for alcoholic beverages) compared with the overall scores. This shows that even for those food groups for which significant correlations were estimated, possible underestimation of dietary intake cannot be excluded. Also, differences across food groups show that purchases are a reasonable reflection of the participants’ reported consumed intake only for some food groups. A stronger correlation for alcohol may mean that most participants consumed alcoholic beverages primarily purchased at the participating supermarket and less so from other retailers. Lastly, although food groups were classified differently in the study by Parker *et al.* (2021), the strength of the association was comparable for fish, nuts and legumes^(13)^.

Lastly, sensitivity analyses showed sex and educational level may play a role in the relationship between purchases and consumption. With regard to sex differences, females may be more likely to underreport than males resulting in a slightly lower correlation^(24)^. Concerning education level, these findings show that the association between purchased and consumed diet quality was stronger among those with lower education. While this variation could be explained by different reasons, if the educational level is interpreted as a proxy for socio-economic status, it is possible that lower-income households may be less accustomed to dining out and more price-sensitive than higher-income (or highly educated) households and might try to produce less food waste by consuming more of the products they had already purchased^(25,26)^. On the other hand, this study also found a stronger association between purchased and consumed diet quality among those with a higher educational attainment compared with those with a medium educational attainment. Several studies have reported a link between higher educational attainment and health consciousness and nutritional knowledge^(27–30)^. This could implicate that those individuals with higher educational attainment might be more inclined to make informed decisions when purchasing food, selecting items that align with their dietary preferences and nutritional goals. They might also be more likely to create shopping lists based on planned meals, ensuring that what they buy aligns closely with what they intend to eat. This could exemplify how people with higher education might have the knowledge and resources to avoid food purchases that are less likely to be consumed.

Future research should validate the relationship between diet quality from purchasing data and reported dietary intake in larger samples, while also assessing the nutritional quality of food purchases. By exploring different measurements of diet quality, future studies could solidify the use of food purchases in epidemiological studies as a proxy for diet quality or as a complement to traditional dietary data collection methods.

In conclusion, the study suggests that dietary intake derived from purchasing data could be a valid and objective method to supplement traditional diet assessment methods in public health research, such as FFQ or dietary recalls, particularly for fruit, nuts, fish and alcoholic beverages. Correlations were stronger in individuals with higher educational attainment. This cost-effective approach may enhance the validity of traditional methods.

## Supporting information

Colizzi et al. supplementary material Colizzi et al. supplementary material

## Supplementary material

For supplementary material/s referred to in this article, please visit https://doi.org/10.1017/S0007114524002630.
