# Supplementary material for: Are food and beverage purchases reflective of dietary intake? Validity of supermarket purchases as indicator of diet quality in the Supreme Nudge Trial
Source: Br J Nutr. 2024 Nov 6;132(10):1394–402. doi: 10.1017/S0007114524002630 (PMC11646671; doi:10.1017/S0007114524002630)
Supplement: Colizzi et al. supplementary material [file S0007114524002630sup001.docx]

**Supplementary Material**

**Are food and beverage purchases reflective of dietary intake? Validity of supermarket purchases as indicator of diet quality in the Supreme Nudge Trial**

Chiara Colizzi, Josine M Stuber, Yvonne T van der Schouw, Joline WJ Beulens

**Supplementary Table 1.** Construction of the purchased and consumed diet quality scores_._

| **Components ***† | **Component Type** † | **Minimum score (= 0 points)** | **Maximum score (=10 points)** |
| --- | --- | --- | --- |
| Vegetables | A | 0 g/d | >= 200 g/d |
| Fruit | A | 0 g/d | >= 200 g/d |
| Whole grains | A | 0 g/d | >= 90 g/d |
| Legumes | A | 0 g/d | >= 10 g/d |
| Nuts | A | 0 g/d | >= 15 g/d |
| Fish | A | 0 g/d | >= 15 g/d |
| Dairy | O | 0 g/d OR >= 750 g/d | 300 – 450 g/d |
| Fats | R | No consumption of healthy fats (=olive oils, sunflower oils, margarines, and vegetable oils) OR ratio of healthy fats to unhealthy fats <= 0·6 | No consumption of unhealthy fats (=butters, baking butters, frying oils, coconut oils, butters, baking butters) OR ratio of healthy fats to unhealthy fats >= 13 |
| Red and Processed Meats | M | >= 50 g/d | 0 g/d |
| SSBs | M | >=250 g/d | 0 g/d |
| Alcohol | M | >= 20 g ethanol/d for women;  >= 30 g ethanol/d for men | <= 10 g ethanol/d for women;  <= 10 g ethanol/d for men |
| Tea and Coffee | Q | > 0 g/d of unfiltered coffee products, tea bags with added sugar, coffee with added sugar or full-fat dairy | >=450 g/d of tea bags, filtered coffee products, coffee products with semi-skimmed and skimmed dairy |
| Snacks | M | >=250 g/d | 0 g/d |

* Components as described by Looman et al. (2017) ^(19)^ except for red meat, tea and coffee, salt (not included), and snacks.

† A = adequacy component; O = optimum component; M = moderation component; R = ratio component; Q = quality component

**Supplementary Table 2.** Percentages used as percentage multipliers to adjust for grocery shopping done at food retailers other than the participating supermarkets at least once in the past two weeks.

| **Type of store** | **% of shoppers** |
| --- | --- |
| Greengrocer | 25% |
| Butcher | 26% |
| Fish shop | 41% |
| Bakery | 52% |
| All other food retailers * | 93% |

* Average from percentage of grocery shopping at least once in the last two weeks at other supermarkets, online supermarkets, local markets, and farmer’s market.

**Supplementary Table 3.** Baseline purchased diet quality, without percentage multipliers as adjustment for shopping in other stores (n=227).

| **Food consumption *** |  |  |
| --- | --- | --- |
| Purchased diet quality, total, scored 0 (low adherence) to 10 (high adherence) | 53 | 17 |
| Purchased diet quality, by sub-components, scored 0 (low adherence) to 10 (high adherence), mean (SD) |  |  |
| Vegetables | 4 | 1 – 9 |
| Fruit | 3 | 1 – 9 |
| Whole grains | 2 | 0 – 5 |
| Legumes | 0 | 0 – 10 |
| Nuts | 0 | 0 – 0 |
| Dairy | 0 | 0 – 5 |
| Fish | 0 | 0 – 8 |
| Added fats | 0 | 0 – 1 |
| Tea and Coffee | 0 | 0 – 10 |
| Red and Processed meat | 4 | 0 – 8 |
| SSBs | 8 | 3 – 10 |
| Alcohol | 10 | 0 – 10 |
| Snacks | 9 | 6 – 10 |

* Expressed as median and interquartile range, unless stated otherwise.

**Supplementary Table 4.** Grams of food purchased (n=227), unadjusted and adjusted for shopping in other stores.

|  | **Food Consumption (grams/day) *** | |
| --- | --- | --- |
|  | Grams of food purchased (unadjusted) | Grams of food purchased (adjusted) |
| Vegetables | 82 (26 – 172) | 80 (27 – 177) |
| Fruit | 63 (14 – 188) | 64 (14 – 186) |
| Whole grains | 14 (0 – 46) | 15 (0 – 57) |
| Legumes | 0 (0 – 11) | 0 (0 – 21) |
| Nuts | 0 (0 – 0) | 0 (0 – 0) |
| Dairy | 170 (64 – 377) | 310 (121 – 679) |
| Fish | 0 (0 – 12) | 0 (0 – 13) |
| Unsaturated fats | 0 (0 – 18) | 0 (0 – 34) |
| Saturated fats | 0 (0 – 9) | 0 (0 – 17) |
| Tea and Coffee (filtered) | 0 (0 – 9) | 0 (0 – 14) |
| Tea and Coffee (unfiltered) | 0 (0 – 0) | 0 (0 – 0) |
| Red and Processed meat | 28 (8 – 79) | 29 (9 – 79) |
| SSBs | 54 (0 – 179) | 68 (0 – 208) |
| Alcohol | 0 (0 – 71) | 0 (0 – 105) |
| Snacks | 35 (8 – 101) | 43 (9 – 127) |

* Expressed as median and interquartile range, unless stated otherwise.

**Supplementary Table 5.** Pearson’s and Spearman rank’s correlation coefficients between purchased and consumed diet qualities, without adjustment for shopping at other stores, overall and by score component (*n*=227).

| **Correlation Coefficient ρ (95% CI)** | |
| --- | --- |
| *Overall* † | 0·31 (0·19, 0·42) * |
| *Score Components* ‡ |  |
| Vegetables | 0·06 (-0·08, 0·19) |
| Fruit | 0·15 (0·02, 0·28) * |
| Whole grains | 0·12 (-0·01, 0·25) |
| Nuts | 0·20 (0·07, 0·33) ** |
| Legumes | 0·05 (-0·11, 0·20) |
| Dairy | 0·21 (0·07, 0·34) ** |
| Fish | 0·17 (0·03, 0·31) * |
| Red and processed meat | 0·18 (0·04, 0·31) ** |
| Fats | -0·04 (-0·19, 0·10) |
| Tea and Coffee | -0·04 (-0·17, 0·10) |
| SSBs | 0·22 (0·08, 0·34) ** |
| Snacks | 0·01 (-0·17, 0·18) |
| Alcohol | 0·39 (0·26, 0·50) ** |

† Calculated using Pearson’s correlation

‡ Calculated using Spearman’s rank correlation

* Significant at 0.05

** Significant at 0.01
